# Neural circuitry governing anxious individuals’ mis-allocation of working memory to threat

**DOI:** 10.1038/s41598-017-08443-7

**Published:** 2017-08-18

**Authors:** Daniel M. Stout, Alexander J. Shackman, Walker S. Pedersen, Tara A. Miskovich, Christine L. Larson

**Affiliations:** 1 Center of Excellence for Stress and Mental Health, VA San Diego Healthcare System, San Diego, CA 92161 USA; 2 Department of Psychiatry, University of California San Diego, San Diego, CA 92093 USA; 3 Department of Psychology, Neuroscience and Cognitive Science Program, and Maryland Neuroimaging Center, University of Maryland, College Park, College Park, MD 20742 USA; 4 Department of Psychology, University of Wisconsin-Milwaukee, Milwaukee, WI 53211 USA

## Abstract

Dispositional anxiety is a trait-like phenotype that confers increased risk for a range of debilitating neuropsychiatric disorders. Like many patients with anxiety disorders, individuals with elevated levels of dispositional anxiety are prone to intrusive and distressing thoughts in the absence of immediate threat. Recent electrophysiological research suggests that these symptoms are rooted in the mis-allocation of working memory (WM) resources to threat-related information. Here, functional MRI was used to identify the network of brain regions that support WM for faces and to quantify the allocation of neural resources to threat-related distracters in 81 young adults. Results revealed widespread evidence of mis-allocation. This was evident in both face-selective regions of the fusiform cortex and domain-general regions of the prefrontal and parietal cortices. This bias was exaggerated among individuals with a more anxious disposition. Mediation analyses provided compelling evidence that anxious individuals’ tendency to mis-allocate WM resources to threat-related distracters is statistically explained by heightened amygdala reactivity. Collectively, these results provide a neurocognitive framework for understanding the pathways linking anxious phenotypes to the development of internalizing psychopathology and set the stage for developing improved intervention strategies.

## Introduction

Heightened levels of dispositional anxiety confer increased risk for the development of internalizing disorders, including anxiety and co-morbid depression^1^. These debilitating psychiatric disorders are common and existing treatments are inconsistently effective^2, 3^, underscoring the need to develop a deeper understanding of the mechanisms governing individual differences in risk. Dispositional anxiety is a trait-like phenotype that first emerges early in life, persists into adulthood, and reflects a combination of heritable and non-heritable factors^1^. Like many patients with anxiety disorders, individuals with elevated levels of dispositional anxiety are prone to pervasive distress and intrusive thoughts in the absence of immediate threat^4, 5^. Recent electrophysiological research motivates the hypothesis that these symptoms partially reflect the mis-allocation of working memory (WM) resources to threat-related cues, promoting rumination about the past and worry about the future^6, 7^. Like selective attention^8^, WM is a limited-capacity workspace where information is transiently stored and used to guide goal-directed cognition^9^. Once lodged in WM, threat-related information is poised to bias thoughts, feelings, and behavior when it is no longer present in the external environment^10, 11^. Although a number of studies have examined ‘emotional’ WM^12, 13^, the neural circuitry underlying mis-allocation of emotional WM, a key feature of the anxious phenotype, has never before been examined using neuroimaging techniques and remains unknown. Addressing this question is important for understanding the mechanisms that confer risk for psychopathology, for optimizing interventions, and for clarifying the biological bases of dispositional anxiety.

**Figure 1:**
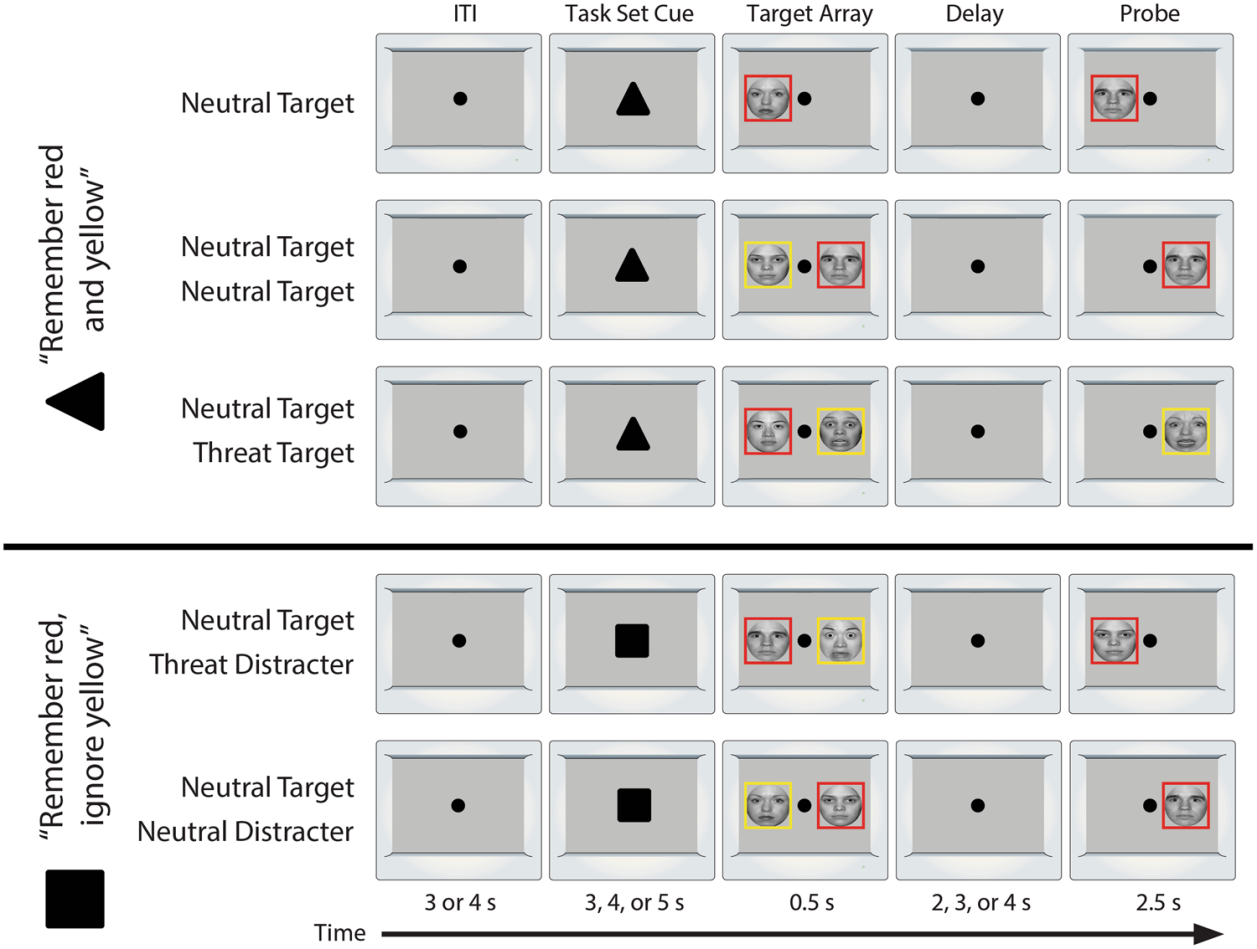
Emotional WM task. Rows depict conditions. Each trial began with a fixation dot, followed by a task set cue. Participants were instructed to either remember all face stimuli (triangle cue; top three rows), or to remember only the target face outlined in red and ignore the distracter face outlined in yellow (square cue; bottom two rows; colors counterbalanced across subjects). Next, the memory array was briefly presented (0.5 s). Following a variable-duration delay (2–4 s), a single probe face was presented. Subjects were instructed to identify whether the target face changed identity or not (equiprobable). On change trials, the identity of the model changed, whereas the expression (fearful or neutral) did not. Trials were separated by a 3–4 s inter-trial interval (ITI). Portions of this figure were adapted with permission from Macmillan Publishers Ltd: Nature Reviews Neuroscience, ref. 72.

Building on prior behavioral and electrophysiological work, functional MRI (fMRI) was used in the present study to quantify neural activity while subjects performed a well-established emotional WM task^6, 7^. As described in more detail in the Method section, subjects were instructed to selectively retain one or two target faces while ignoring distracters (Fig. 1). Target and distracter face stimuli displayed either threat-related (i.e., fearful) or emotionally neutral expressions^14^. Subjects also performed a separate ‘localizer’ task^15^, enabling us to independently identify regions involved in face perception. Using these data, we tested whether WM resources are mis-allocated to threat-related distracters and the degree to which this is enhanced among individuals with a more anxious disposition. To enhance reproducibility and power^16^, hypothesis testing focused on three *a priori* regions of interest (ROIs): domain-general WM regions in the posterior parietal cortex (PPC) and dorsolateral prefrontal cortex (dlPFC) and a domain-selective cache in the fusiform face area (FFA). The PPC and dlPFC both play a key role in biasing attention and facilitating WM for a broad range of visual stimuli^9, 17^. In contrast, the FFA plays a central role in face perception^18^ and selectively supports the short-term retention of faces^19^. Applying standard approaches developed by Vogel and McNab for emotionally neutral stimuli^20, 21^, mis-allocation scores were separately computed for threat-related and neutral distracters by taking the difference in activation between distracter-present and -absent trials. Scores greater than zero reflect the mis-allocation of WM to the distracter. Secondary analyses directly compared threat-related to neutral distracters.

Additional analyses tested whether the mis-allocation of WM resources to threat reflects a downstream consequence of heightened amygdala reactivity, as we previously hypothesized^7^. The amygdala is known to play a key role in biasing attention to threat-related information and is more reactive to threat among individuals with elevated levels of dispositional anxiety or anxiety disorders^22^. Here, a statistical mediation framework was used^23^ to rigorously test whether anxious individuals’ biased allocation of WM resources can be explained by heightened amygdala reactivity to threat.

## Results

### WM is mis-allocated to threat-related distracters

**Figure 2:**
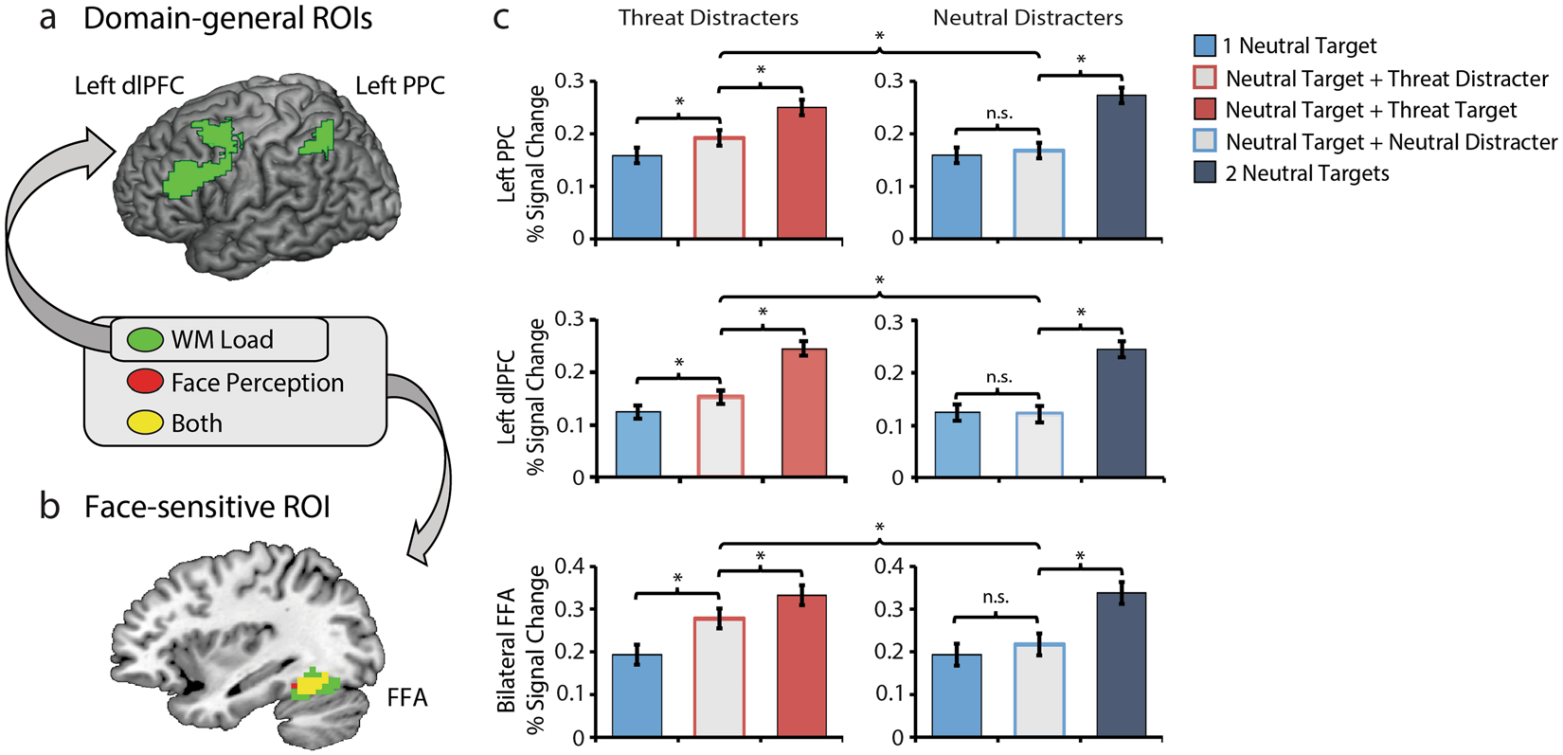
WM is mis-allocated to threat-related distracters. To enhance reproducibility and power^16^, hypothesis testing focused on several key WM regions. Each ROI was defined using a combination of anatomical and functional criteria. (**a**) *Domain-general ROIs*. WM load-sensitive voxels within the dlPFC (MFG) and PPC (IPL) are depicted in green (*p*s < 0.05, corrected). (**b**) *Face-sensitive ROI*. Voxels sensitive to both WM load (green) and face perception (red; indexed using data from the independent ‘localizer’ task) within the FFA (temporal-occipital fusiform cortex) are depicted in yellow (*p* < 0.05, corrected). **(c)** Percentage signal change for each condition. As shown on the left side of panel c, activation in the left PPC (top row), left dlPFC (middle row), and bilateral FFA (bottom row) exhibited a monotonic, step-like pattern of activation across conditions, indicating partial mis-allocation of WM resources to the threat-related distracter. As shown on the right side of panel c, the presence of an emotionally neutral distracter was not sufficient to significantly alter activation. Asterisks indicate *p* < 0.05. Error bars indicate the nominal probability of the null hypothesis being rejected by chance: *p* < 0.05 (non-overlapping bars) or *p* > 0.05 (overlapping bars) and were computed as described in ref. 73. Abbreviations—Dorsolateral Prefrontal Cortex (dlPFC), Fusiform Face Area (FFA), Inferior Parietal Lobule (IPL), Middle Frontal Gyrus (MFG), Posterior Parietal Cortex (PPC).

If threat-related distracters more readily infiltrate WM, we would expect load-sensitive regions of the PPC, dlPFC, and FFA to track this mis-allocation of resources (Fig. 2a,b). As shown in Fig. 2c, analyses revealed that the presence of a single threat-related distracter is associated with significantly greater activation in all three ROIs compared to distracter-absent (i.e., one-target) trials, *t*s(80) > 2.16, *p*s < 0.03. The monotonic, step-like pattern of activation across the three conditions (Target < Target + Threat-Related Distracter < Two Targets) indicates that threat-related distracters did not completely consume WM. Indeed, activation in the left PPC, left dlPFC, and FFA was significantly greater for two-target trials, *t*s(80) > −2.20, *p*s < 0.03. Mis-allocation effects were weaker in the right PPC and right dlPFC for both threat-related and neutral distracters (*t*s(80) < 1.61, *p*s > 0.11).

WM mis-allocation was enhanced for threat distracters. As shown in Fig. 2c, the threat-related distracter was associated with more activation than the neutral distracter in all three regions, *t*s(80) > 2.09, *p*s < 0.04. In contrast, activation in the one-target and neutral-distracter conditions did not differ (Target ≅ Target + Neutral Distracter), *t*s(80) < −0.74, *p*s > 0.46, indicating that subjects were much less susceptible to allocating WM to neutral distracters.

Other analyses indicated that the mis-allocation of WM to threat-related distracters was not an artifact of expression-dependent differences in storage. As shown in Fig. 2c (dark red and dark blue bars), similar levels of activation were evident in all three regions during the maintenance of threat-related and neutral facial expressions when they were task-relevant targets (2 Neutral Targets ≅ Neutral Target + Threat Target), *t*s(80) < 1.22, *p*s > 0.22.

Collectively, these findings demonstrate that WM resources in the PPC, dlPFC, and FFA are mis-allocated to threat-related information when it is irrelevant to on-going task demands, extending the results of earlier electrophysiological research^7^.

### Dispositional anxiety is associated with increased mis-allocation of WM to threat-related distracters

**Figure 3:**
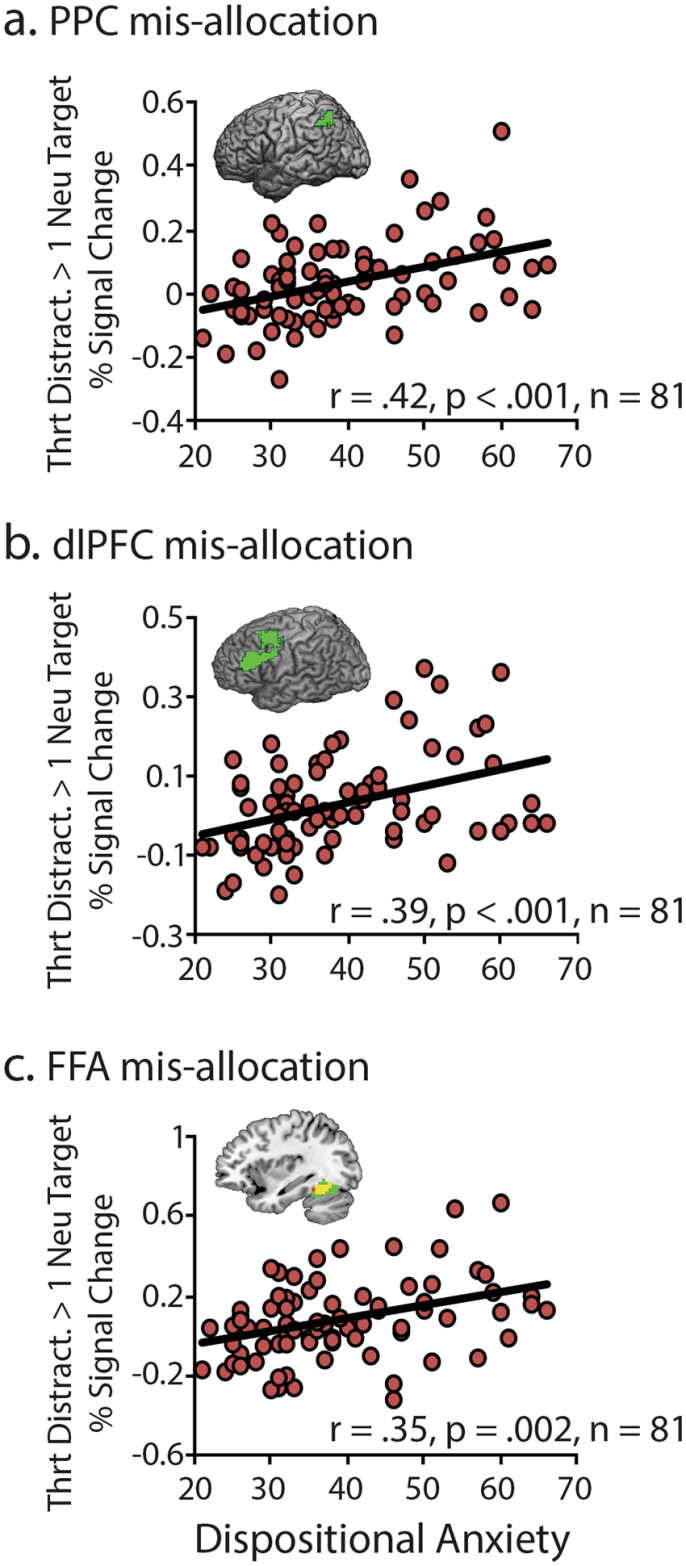
Dispositional anxiety predicts the mis-allocation of WM to threat-related distracters. Mis-allocation scores were defined as the difference in activation associated with the presence of a distracter (e.g., Neutral Target + Threat-Related Distracter minus Neutral Target). Larger scores indicate greater allocation of WM resources to threat-related distracters. Analyses revealed that higher levels of dispositional anxiety are associated with increased mis-allocation of WM to threat-related distracters in the (**a**) PPC, (**b**) dlPFC, and (**c**) FFA ROIs.

To test whether dispositionally anxious individuals allocate more WM resources to threat-related distracters, mis-allocation scores were computed. As in prior electrophysiological^21, 24^ and imaging work^20^, scores were computed by taking the difference in activation between distracter-present and distracter-absent (i.e., one-target) trials. As described in more detail in the Method section, positive scores reflect the mis-allocation of WM resources to the distracter. As shown in Fig. 3, analyses revealed that dispositionally anxious individuals mis-allocated more domain-general (left PPC: *r* = 0.42, *p* < 0.001; left dlPFC: *r* = 0.39, *p* < 0.001) and face-sensitive (FFA: *r* = 0.35, *p* = 0.002) resources to threat-related distracters. These relations remained after controlling for nuisance variation in age, sex, and working memory capacity (*pr*s > 0.36, *p*s < 0.001). In a series of simultaneous regressions, the mis-allocation of WM to threat-related distracters predicted dispositional anxiety (*pr*s > 0.26, *p*s < 0.02), whereas the mis-allocation to neutral distracters was numerically weaker and nonsignificant (*pr*s < 0.15, *p*s > 0.19). Moreover, relations with dispositional anxiety were only observed when threat-related facial expressions were task-irrelevant. That is, dispositional anxiety was not significantly related to activation associated with either threat-related or neutral faces when they were targets (Threat: *r*s < 0.20, *p*s > 0.07; Neutral: *r*s < 0.11, *p*s > 0.31). Taken together, these findings provide evidence that elevated levels of dispositional anxiety are associated with the indiscriminate allocation of both domain-general and face-sensitive WM resources to threat-related distracters.

### Heightened amygdala engagement promotes the mis-allocation of WM to threat-related distracters

**Figure 4:**
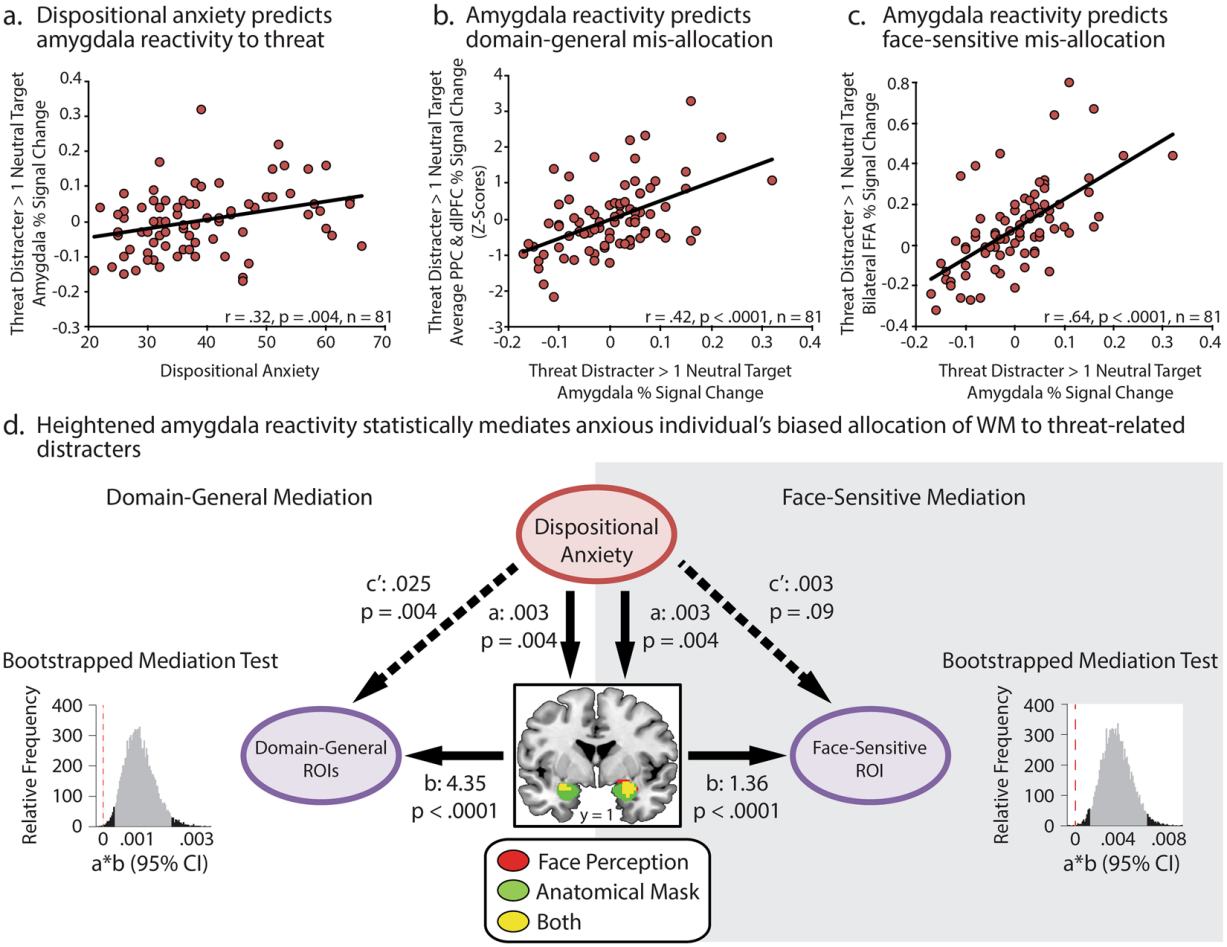
The amygdala promotes the mis-allocation of WM to threat-related distracters. (**a**) Individuals with a more anxious disposition show elevated amygdala reactivity to threat-related distracters. (**b**,**c**) In turn, elevated amygdala reactivity is associated with increased mis-allocation of domain-general (left PPC and left dlPFC) and face-sensitive (FFA) WM. (**d**) Path models and bootstrapping results are depicted for domain-general (left PPC and left dlPFC) and face-sensitive (FFA) ROIs. In both, relations between anxiety and WM mis-allocation were significantly reduced (path *c*’) after controlling for threat-related amygdala activation, indicating significant mediation. Histograms show the bootstrapped 95% CI for the mediation test (product of paths *a* and *b*; 10,000 samples). The 95% CIs are depicted in gray, with the dotted red line indicating zero.

Next, a multivariate mediation approach was used^25, 26^ to test whether the association between individual differences in dispositional anxiety and WM mis-allocation reflects heightened amygdala reactivity to threat-related distracters (i.e., Dispositional Anxiety → Amygdala → WM Mis-allocation; Fig. 4), as we previously hypothesized^7^. Separate analyses were performed for the average of the two domain-general ROIs (i.e., mean of *z*-transformed mis-allocation scores for the left PPC and left dlPFC; see the Supplement) and for the face-sensitive FFA ROI. As shown in Fig. 4a, analyses showed that dispositionally anxious individuals exhibit heightened reactivity in the amygdala to threat-related distracters (*r* = 0.32, *p* = 0.004), but not neutral distracters (*r* = 0.13, *p* = 0.25). Heightened amygdala activation to threat was, in turn, positively associated with WM mis-allocation in both domain-general (*r* = 0.51, *p* < 0.0001; Fig. 4b) and face-sensitive ROIs (*r* = 0.66, *p* < 0.0001; Fig. 4c). Finally, a non-parametric bootstrapping approach^26^ was used to show that heightened amygdala reactivity significantly mediates the association linking dispositional anxiety to the mis-allocation of WM resources (Sobel’s *p*s < 0.022; Fig. 4d). A potential concern with this result is that the three WM-related ROIs were defined using ‘non-independent’ data (i.e., the load contrast from the experimental task), which has the potential to distort effect estimates^27^. As detailed in the Supplementary Results, both mediation analyses remained significant when we substituted ROIs that were independently defined in a completely independent manner using the results of an automated WM meta-analysis performed using NeuroSynth^28^. Collectively, these findings provide compelling evidence that anxious individuals’ tendency to mis-allocate WM to threat-related distracters is a downstream consequence of heightened amygdala reactivity.

### Performance

**Figure 5:**
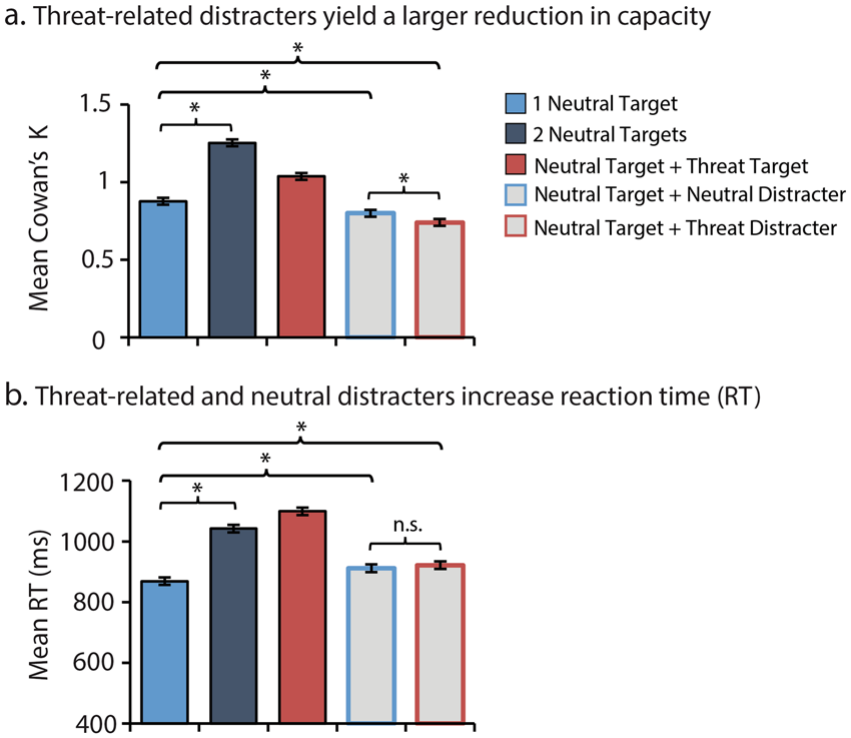
Influence of load and distracters on behavior. (**a**) WM capacity (*K*) scales with load and is reduced by distracters. Trials with a threat-related distracter (Neutral Target + Threat Distracter) were associated with a greater reduction in storage capacity than trials with a neutral distracter (Neutral Target + Neutral Distracter) (**b**) Reaction time (RT) scales with WM load and is increased by both threat and neutral distracters to a similar degree. Asterisks indicate *p* < 0.05. Error bars indicate the nominal probability of the null hypothesis being rejected by chance: *p* < 0.05 (non-overlapping bars) or *p* > 0.05 (overlapping bars) and were computed as described in ref. 73.

As shown in Fig. 5a, an omnibus analysis confirmed that WM capacity (*K*) increased from one to two neutral target faces (*p* < 0.001). WM capacity was impaired by both threat-related (*p* < 0.001) and neutral distracters (*p* < 0.001), but the impairment was greater for threat compared to neutral distracters (*p* = 0.001). Reaction time (RT) (Fig. 5b) analyses revealed a similar pattern. Performance was slower at the higher WM load or in the presence of distracters (*p*s < 0.001. In contrast to *K*, RT did not differ between threat-related and neutral distracters (*p* = 0.21). Individual differences in dispositional anxiety were not significantly related to either performance metric (*p*s > 0.46).

## Discussion

Like many patients with anxiety disorders, individuals with an anxious disposition are prone to intrusive thoughts and heightened distress in the absence of immediate danger^1^. Recent electrophysiological research suggests that this reflects the mis-allocation of WM resources to threat-related information, promoting negative mood, rumination, and worry^7^. The present study provides new insights into the neural systems governing this mnemonic deficit, demonstrating that both domain-general (PPC and dlPFC) and face-selective WM resources (FFA) are mis-allocated to threat-related distracters (Fig. 2) and that this bias is exaggerated among individuals with a more anxious disposition (Fig. 3). Importantly, multivariate mediation analyses provided evidence that anxious individuals’ biased allocation of WM resources reflects heightened amygdala reactivity to threat (Fig. 4). Threat was associated with excess activation in the PPC, dlPFC, and FFA compared to either distracter-absent or neutral-distracter trials, but *only* when it was task-irrelevant and there was an opportunity to mis-allocate WM resources. In contrast, threat-related and neutral expressions elicited comparable levels of activation in these regions when they were relevant to the WM task. Likewise, dispositional anxiety was associated with the mis-allocation of WM resources to threat-related distracters, but *not* with the allocation of resources to neutral distracters or threat-related targets. Collectively, these observations show that dispositionally anxious individuals show a bias to allocate WM resources to threat-related distracters.

The results of our mediation analyses suggest that the amygdala promotes the mis-allocation of WM resources to threat-related distracters. The amygdala is sensitive to a broad spectrum of emotionally salient stimuli, including threat-related facial expressions^29, 30^. In addition, there is clear evidence that anxious individuals show amplified or prolonged amygdala responses to threat-related faces^1^, even when they are task-irrelevant^31^, consistent with our results (Fig. 4a). Anatomically, the amygdala is well positioned to prioritize the short-term retention of threat-related cues. This may occur *indirectly*, via projections from the amygdala to neuromodulatory systems in the basal forebrain and brainstem that are poised to influence the quality of neuronal signal processing (e.g., signal-to-noise ratio) throughout the brain or *directly*, via excitatory projections from the amygdala to the FFA, PPC, and other regions of visual cortex^32–34^. Indeed, mechanistic work in humans and monkeys demonstrates that the amygdala plays a crucial role in amplifying the processing of threat-related faces in the ventral visual processing stream, including the FFA^35, 36^. Other work indicates that the amygdala plays a key role in triggering reflexive shifts in attention to threat-diagnostic facial features, such as the eyes and brow, via projections to the superior colliculus and other regions^37, 38^. This bias is exaggerated among anxious individuals^39, 40^ and would plausibly account for the specificity of our results to trials in which threat is irrelevant to on-going task demands. In short, while our results provide compelling evidence that anxious individuals mis-allocate WM resources to threat, an important challenge for future research will be to determine whether this deficit reflects shifts in overt or covert attention to threat-related distracters at the time of encoding.

Dispositional anxiety is a prominent risk factor for the internalizing disorders^1^ and our results provide a framework for understanding the origins of this liability. Like patients with mood and anxiety disorders, individuals with an anxious disposition are prone to intrusive and distressing thoughts, worries, and memories^5^. The biased allocation of WM resources to threat may help to explain these symptoms. That is, once resident in WM, threat-related information is poised to exert a persistent bias on mood, cognition, and behavior^10, 11^, promoting affective inertia and mood ‘spill-over’ across sequential moments and contexts^41, 42^. This framework also provides an attractive explanation for the intrusive memories that are characteristic of extreme dispositional anxiety and many disorders on the internalizing spectrum^43^. In particular, it has become clear that information can enter WM via either perceptual encoding or retrieval from long-term memory (LTM;^44^. From this perspective, WM reflects the temporary allocation of selective attention to recently perceived items or the temporary re-activation of representations stored in LTM^45, 46^. This suggests that intrusive memories may reflect the mis-allocation of WM resources to distressing material held in LTM.

Our observations also provide a framework for understanding the amygdala’s contribution to the etiology of anxiety and mood disorders. Like elevated levels of dispositional anxiety^22^, increased amygdala reactivity to threat-related cues confers increased risk for the development of future internalizing symptoms, characterizes patients with internalizing disorders, and is normalized by clinically effective treatments for anxiety and depression^47, 48^. Mechanistic work in rodents and monkeys indicates that the amygdala is a critical component of the distributed circuitry underlying extreme anxiety^49, 50^, consistent with observations of humans with naturally occurring amygdala damage^51^. The present results extend this line of work, demonstrating that heightened amygdala reactivity also promotes the retention of threat-related information in WM. An important avenue for future research will be to test whether this WM bias explains the sustained distress and intrusive thoughts that characterize many patients with anxiety and mood disorders.

Existing treatments for the internalizing disorders are inconsistently effective or associated with significant adverse effects^52^, underscoring the need to develop more effective intervention strategies. The present results highlight the utility of therapeutics targeting the amygdala. Targeting upstream regions that are poised to regulate the amygdala might also prove useful. In this regard, cognitive-behavioral therapy, which is thought to be mediated by emotion regulatory processes implemented in the prefrontal cortex^53, 54^ may be particularly effective. Cognitive remediation and computerized adaptive training techniques, including recently established ‘closed-loop’ neurofeedback strategies, represent another potential means of short-circuiting the mis-allocation of WM resources^55^. Research focused on such interventions would also provide an important opportunity to gain mechanistic insight into the neural circuitry underlying the biased allocation of WM to threat.

Clearly, important challenges remain. Adapting paradigms to include a longer delay interval may prove helpful in determining the chronometry and extent to which threat-distracters are maintained in WM, differentiate between attention allocation and WM maintenance processes, and clarify how these can influence other key anxiety-related symptoms (e.g., avoidant behavior, retrieval of negative memories). It will also be useful to extend our approach to learned threats, which would enable the use simpler geometric cues (e.g., colored bars, circles) and allow a more direct comparison with other work in the cognitive neurosciences focused on WM mis-allocation (e.g. refs 20, 21, 56). Absent such comparisons, the generalizability of our results to other kinds of threat remains unclear. Another potential limitation is the absence of an anxiety-behavior relationship. However, according to the attentional control theory^57^, anxious individuals can perform effectively by increasing attentional control to compensate for excess WM load by irrelevant information^58, 59^. Our finding of an anxiety-related increase of the WM network but no relationship with behavior is consistent with this theory. Finally, dispositional anxiety was operationally defined by using the STAI. However, the STAI is not specific to anxiety, but instead measures general negative affect, behavior, and cognition^4, 60^. Therefore, the current findings likely generalize to the broader concept of dispositional negativity, and the attendant vulnerability to anxiety, mood, and substance use disorders^1^.

## Conclusions

Collectively, the present results demonstrate that WM resources are mis-allocated to threat-related information when it is irrelevant to on-going task demands. This WM bias is exaggerated among more anxious individuals and appears to reflect heightened amygdala reactivity to threat. The use of a relatively large sample, hypothesis-driven analytic strategy, and well-established emotional WM task enhances confidence in the reproducibility and translational significance of our results. More broadly, these observations provide a framework for understanding the neurocognitive processes underlying variation in dispositional anxiety and set the stage for mechanistic studies aimed at identifying more effective intervention strategies for the internalizing spectrum of disorders.

## Method

### Subjects

A total of 105 right-handed individuals were imaged. Twenty-four subjects were excluded for technical problems (*n* = 2), below-chance performance (*n* = 6), or excessive motion artifact (>3 mm; *n* = 16). Included and excluded subjects did not significantly differ in dispositional anxiety, *p* = 0.23. After exclusion, 81 subjects remained for primary analyses (Supplementary Table 1). This sample size provides 78.6% power for detecting a ‘medium-sized’ (*r* = 0.30)^61^ association between dispositional anxiety and brain function (two-tailed α = 0.05; bivariate normal model; exact test; G * Power version 3.1.9.2), more than twice that of a typical imaging study (35.9% power based on an estimated median sample size of *n* = 28.5)^16^. A subset of subjects (*n* = 77) provided usable data for the face ‘localizer’ task. The study protocol and procedures were approved by the Institutional Review Boards at the University of Wisconsin-Milwaukee and the Medical College of Wisconsin. The study was conducted in accordance with their guidelines and regulations involving human research subjects. All subjects provided informed written consent prior to participating.

### Dispositional anxiety

As in prior work^6, 7^, the State-Trait Anxiety Inventory (STAI; α = 0.94) was used to quantify individual differences in dispositional anxiety (Supplementary Table 1)^62^.

### Emotional WM task

The emotional WM task was adapted from prior electrophysiological and behavioral research^6, 7, 20^. The task employed equiluminant, gray-scale faces extracted from the Ekman^63^ and NimStim (https://www.macbrain.org) databases. Trial sequence and timing were derived from work by McNab and Klingberg^20^. As shown in Fig. 1, trials began with a fixation cross (3–4 s), followed by a task-set cue (3–5 s). Participants were instructed to either remember all face stimuli (black triangle cue; top three rows of Fig. 1) or remember the target face outlined in red while ignoring the distracter face outlined in yellow (black square cue; bottom two rows in Fig. 1; colors counterbalanced across subjects). Next, the memory array was presented for 0.5 s followed by a delay period (2–4 s). Finally, a single probe face was presented (2.5 s). Subjects were instructed to identify whether the target face changed identity or not (equiprobable). On change trials, the target face was replaced with a new face, but the expression remained unchanged. The task included trials with threat-related distracters (1 Neutral Target +1 Threat-Related Distracter) or neutral distracters (1 Neutral Target +1 Neutral Distracter). The distracter trials allowed us to test mis-allocation of working memory storage to task-irrelevant information. In order to functionally define load-sensitive ROIs (see below), the task also incorporated trials with two targets (i.e., 1 Neutral Target +1 Neutral Target and 1 Neutral Target +1 Threat-Related Target), and one target (1 Neutral Target). Subjects performed 20 practice trials before completing 140 experimental trials (pseudo-randomized, event-related design; 40 trials/distracter condition; 20 trials/target-only condition) across four scans.

### Face ‘localizer’ task

In order to define face-sensitive ROIs (see below) in an unbiased manner, subjects completed an independent face ‘localizer’ task, where they passively viewed face or house images (20 600-msec images/block; 12 blocks/condition). The task employed equiluminant, gray-scale faces from the FERET database^64^ and house stimuli from prior studies^65^.

### MRI data acquisition and preprocessing

MRI data were acquired using a General Electric 3 T scanner and 8-channel head-coil (Waukesha, WI). *T*_1_-weighted anatomical images were acquired using a spoiled gradient-recalled echo sequence (inversion time/repetition time/echo time/flip angle/field of view/matrix/slice thickness: 450 ms/8.2 ms/3.2 ms/12°/240 mm/256 × 224/1 mm). Functional scans were obtained using a T_2_*-weighted echo-planar image (EPI) sequence (repetition time/echo time/flip angle/number of excitations/field of view/matrix/slice thickness [orientation]/slices/gap/volumes: 2000 ms/25 ms/77°/1/240 mm/64 × 64/3.5 mm^3^ sagittal]/41/0 mm/242 volumes for each of four WM scans; 133 volumes for the face ‘localizer’ scan).

The first 3 volumes of each EPI scan were discarded and the remaining volumes were slice-time and motion corrected using AFNI^66^. T_1_-weighted datasets were registered to the MNI152 template using FLIRT and FNIRT (http://fsl.fmrib.ox.ac.uk). Transformation matrices were concatenated and applied to the EPI data in a single step. Datasets were visually inspected for quality assurance. EPI data were spatially smoothed (4-mm FWHM) and converted to percent signal change.

### fMRI modeling

For the WM task, the two cues, five target-arrays, and probe were modeled using separate regressors (Fig. 1) convolved with a canonical hemodynamic response function. As in prior work focused on WM mis-allocation^20^, storage-related activity was modeled as a variable-duration boxcar spanning the array and delay (2.5–4.5 s). For the ‘localizer’ task, face blocks were modeled using a 12-s boxcar. For both tasks, nuisance variates included drift (linear, quadratic, and cubic) and motion (L/R, A/P, S/I, roll, pitch, yaw, and first derivatives) terms. As in prior research^20^, trials with incorrect responses were censored (*M* = 86% trials retained, *SD* = 12%). The percentage of censored trials did not significantly vary with dispositional anxiety, *p* = 0.30.

### ROI Definitions

To enhance power and reduce bias^16^, hypothesis testing focused on three kinds of WM-related ROIs (PPC, dlPFC, and FFA) and the amygdala (Figs 2 and 4 and Supplementary Table 2). As described in detail below, the four ROIs were defined using a combination of neuroanatomical and functional criteria.

The *PPC ROI* included load-sensitive voxels in the inferior parietal lobule (IPL), which encompasses the intraparietal sulcus, an important, domain-general node in the distributed circuit supporting selective attention and WM^67^. The *dlPFC ROI* included load-sensitive voxels in the middle frontal gyrus (MFG). The *FFA ROI* included load- and face-sensitive voxels in the temporal-occipital fusiform cortex. The *Amygdala ROI* included face-sensitive voxels in the atlas-defined amygdala.

In each case, anatomical masks (e.g., IPL) were probabilistically defined using the Harvard-Oxford atlas^68^, *Load Sensitivity* was defined by significantly greater activation for two neutral targets vs. one neutral target (experimental task), and *Face Sensitivity* was defined by significantly greater activation for faces vs. houses (‘localizer’ task) (*p*s < 0.05, corrected for the volume of the relevant anatomical mask). As in other recent work by our group^69^, statistical maps were thresholded based on cluster extent. Null distributions were estimated via Monte Carlo simulations (10,000 iterations) using *3dClustSim*. Simulations incorporated the mean spatial smoothness of the single-subject residuals, estimated using *3dFWHMx*, and a cluster-defining threshold of *p* = 0.005. Preliminary analyses revealed similar effects in the left and right FFA and the left and right amygdala. Therefore, analyses were collapsed across hemispheres using bilateral ROIs.

### Hypothesis testing strategy

#### Mean differences analyses

As in prior work focused on emotional faces^6, 7^ and simpler geometric cues^20, 24^ in order to understand the degree to which WM is mis-allocated to threat, mean percent signal change between the distracter-present condition (1 Neutral Target +1 Threat-Related Distracter) and the distracter-absent condition (1 Neutral Target) was extracted from each WM-related ROI (PPC, dlPFC, and FFA) and compared. Parallel analyses were performed for neutral stimuli. To assess the specificity of WM allocation, direct comparisons between the two target conditions were also computed (1 Neutral Target +1 Threat-Related Target vs. 1 Neutral Target +1 Neutral Target).

#### Individual differences analyses

Mis-allocation scores were separately computed for threat-related and neutral distracters by taking the difference in mean percent-signal between distracter-present (e.g., 1 Neutral Target + 1 Threat-Related Distracter) and distracter-absent trials (1 Neutral Target) for each ROI. Positive scores indicate mis-allocation of WM resources to the distracter. Thus, if an individual was perfectly adept at allocating WM resources, then activity will be similar for trials distracter-present (1 Neutral Target + 1 Threat-Related Distracter) and distracter-absent trials (1 Neutral Target). Conversely, if an individual completely mis-allocates WM resources to the distractor, then both the target and the threat-related distracter (1 Neutral Target + 1 Threat –Related Distracter) will be retained in working memory, and activity in WM-related ROIs will be increased compared to distractor-absent trials. Calculation of mis-allocation scores or conceptually similar metrics is standard practice in imaging and electrophysiology studies of of WM^20, 21, 70^. To assess the specificity of relations between dispositional anxiety and WM mis-allocation, follow-up analyses included nuisance variation in mean-centered age, sex, and working memory capacity (i.e., the maximal Cowan’s *K* across distracter-absent conditions).

#### Mediation analyses

A standard multivariate analytic framework was used^23^ to test whether anxious individuals’ tendency to mis-allocate WM to threat-related distracters is statistically mediated by increased amygdala reactivity to threat (i.e., Dispositional Anxiety → Amygdala → WM Mis-allocation), as we previously hypothesized^7^. Satisfying the criteria of this framework would demonstrate that a significant proportion of the association between anxiety and WM mis-allocation reflects amygdala hyper-reactivity^23^, an inference not afforded by simpler bivariate tests. Operationally, mediation requires four significant regressions: (*a*) Anxiety → Amygdala; (*b*) Amygdala → Mis-Allocation, controlling for Anxiety; (*c*) Anxiety → Mis-Allocation; and (*c’*) Anxiety → Mis-Allocation, controlling for Amygdala. Consistent with prior work^26^, the final criterion was assessed using a non-parametric bootstrapping approach (10,000 samples). The more traditional Sobel’s test was also computed. Naturally, these tests do not provide evidence of causation. Mediation analyses were performed using mis-allocation scores extracted from the face-sensitive FFA ROI and for the mean of the two domain-general ROIs (i.e., *z*-transformed left PPC and left dlPFC). Analyses conducted separately for the left PPC and the left dlPFC revealed similar results (see the Supplement).

### Performance

Cowan’s *K* was estimated as *S* × (*H* − *FA*), where *K* is the estimated number of items maintained in WM, *S* is array set-size, *H* is hit-rate, and *FA* is false-alarm rate^71^. Mis-allocation scores were computed as above.

### Data availability

The datasets generated and analyzed during the current study are available in the NeuroVault repository.

## Electronic supplementary material

Supplementary Information
