# Supplementary material for: Neural circuitry governing anxious individuals’ mis-allocation of working memory to threat
Source: Sci Rep. 2017 Aug 18;7:8742. doi: 10.1038/s41598-017-08443-7 (PMC5562789; doi:10.1038/s41598-017-08443-7)
Supplement: Supplementary file 1 — Supplementary Information [file 41598_2017_8443_MOESM1_ESM.doc]

**Supplementary Results to Accompany**

**Neural circuitry governing anxious individuals’**

**mis-allocation of working memory to threat**

DM Stout, AJ Shackman, WS Pedersen, TA Miskovich, & CL Larson

**Please address correspondence to:**

Dr. Christine L. Larson (larsoncl@uwm.edu)

Department of Psychology

University of Wisconsin-Milwaukee

2441 E. Hartford Avenue

Milwaukee, WI 53211 USA

| Supplementary Table 1. Regions of interest (ROI). | | | | | | | | | | | | |
| --- | --- | --- | --- | --- | --- | --- | --- | --- | --- | --- | --- | --- |
|  | Working Memory Load^a^ | | |  | Face Maximum^b^ | | |  | Center of  Mass^c^ | | |  |
| *Region* | *x* | *y* | *z* |  | *x* | *y* | *z* |  | *x* | *y* | *z* | Volume (mm^3^) |
| Left dlPFC | -39 | 22 | 22 |  | - | - | - |  | -37 | 16 | 36 | 333 |
| Left PPC | -33 | -51 | 39 |  | - | - | - |  | -31 | -52 | 46 | 163 |
| Left FFA | -43 | -54 | -18 |  | -43 | -54 | -18 |  | -37 | -54 | -18 | 38 |
| Right FFA | 44 | -51 | -17 |  | 44 | -51 | -17 |  | 40 | -51 | -18 | 26 |
| Left Amygdala | - | - | - |  | -24 | -5 | -17 |  | -23 | -4 | -18 | 37 |
| Right Amygdala | - | - | - |  | 24 | -4 | -17 |  | 24 | -3 | -18 | 49 |
| *^a^*Load contrast (experimental task), *p*<.05 corrected for the volume of the relevant anatomical mask. *^b^*Faces-vs.-Houses contrast (‘localizer’ task), *p*<.05 corrected for the volume of the relevant anatomical mask. *^c^*Center of mass for the ROI used for hypothesis testing. The FFA and amygdala were collapsed across hemisphere for hypothesis testing. Volume is reported for the ROI used for hypothesis testing (in voxels 3.5mm^3^). | | | | | | | | | | | | |

| Supplementary Table 2. Sample demographics (*n* = 81*^a^*). | | |
| --- | --- | --- |
|  | Mean (*SD*) | Range |
| Age*^b^* | 22.5 (4.1) | 18-35 |
| Spielberger State-Trait Anxiety Inventory (STAI), Trait Version | 39.6 (10.9) | 21-66 |
| *^a^*53 females and 28 males. *^b^*years. | | |

**Amygdala activation mediates domain-general working memory mis-allocation**

We computed multivariate mediation analyses for the left PPC and left dlPFC (i.e., the two domain-general working memory caches) considered separately. We confirmed that amygdala reactivity is associated with enhanced working memory misallocation for threat-related distracters in the left PPC (*r*=.51, *p*<.001) and left dlPFC (*r*=.47, *p*<.001). Next, we computed separate non-parametric bootstrapped mediation tests. For both regions, the amygdala significantly mediated the relationship between dispositional anxiety and working memory mis-allocation: tests of the indirect effect did not cross zero for the left PPC (lower/upper boot: .0005/.0042; Sobel’s Z: 2.57, *p* = .01) and the left dlPFC (lower/upper boot: .0005/.0031; Sobel’s Z: 2.37, *p* = .02). For parsimony, we combined the left PPC and left dlPFC ROIs into a single domain-general ROI (*z*-scored and averaged) and describe this in the main report.

**Key results remain significant using WM-related ROIs independently defined in NeuroSynth**

Whole-brain fMRI analyses suffer from low power, due to the need to correct for thousands of voxelwise comparisons, and produce optimistically biased effect-size estimates ^1, 2^. This bias reflects the fact that voxel selection (‘thresholding’) and effect estimation are combined into a single step ^3^. Completely eliminating this bias using *a priori* anatomical ROIs is typically impractical because there is not a one-to-one mapping between brain regions (e.g., gyri or cytoarchitectonic areas) and specific brain functions. For example, there is abundant evidence that only a small portion of the fusiform gyrus is preferentially sensitive to faces (e.g., Supplementary Ref. ^4^).

In the present report, we used several strategies to increase statistical power (sensitivity), reduce bias, and enhance reproducibility ^5-7^.

1. We collected a relatively large sample (*n*=81), nearly three times larger than the typical fMRI study (median *n*=28.5 in 2015 ^2^).
2. We adopted a hypothesis-driven analytic strategy that was derived from previously published predictions. This strategy focused on three kinds of WM-related ROIs (PPC, dlPFC, and FFA) and the amygdala. All four ROIs were defined using a combination of neuroanatomical and functional criteria. Hypothesis testing used the average signal extracted from these the 4 ROIs.

Despite their advantages, we recognize that the three WM-related ROIs were defined using ‘non-independent’ data (i.e., the load contrast from the experimental task), which has the potential to distort effect estimates ^8^. To assess the influence of this choice, we re-computed our key analyses using ROIs that were independently defined using the results of a ‘generic’ WM meta-analysis (901 studies; forward-inference map; *Z*>5.0; FDR *q*<.01) performed in NeuroSynth ^9^. ROIs were otherwise defined as described in the main report. Analyses demonstrated that anxious individuals mis-allocated more domain-general (left PPC: *r*=.40, *p*<.001; left dlPFC: *r*=.36, *p*=.001) and face-sensitive (FFA: *r*=.26, *p*=.02) resources to threat-related distracters. Moreover, heightened amygdala activation to threat was positively associated with WM mis-allocation in both domain-general (*r*=.56, *p*<.001) and face-sensitive ROIs (*r*=.51, *p*<.001) and significantly mediated the association linking dispositional anxiety to the mis-allocation of WM resources (Sobel’s *p*s<.02; bootstrapped confidence intervals did not include 0). In sum, all of the critical individual differences and mediation results remained significant using ROIs that were defined independently of the experimental task.

**Supplementary References**

1. Kriegeskorte, N., Lindquist, M. A., Nichols, T. E., Poldrack, R. A. & Vul, E. Everything you never wanted to know about circular analysis, but were afraid to ask. *J. Cerebr. Blood F. Met.* **30,** 1551-1557 (2010).

2. Poldrack, R. A. *et al*. Scanning the horizon: towards transparent and reproducible neuroimaging research. *Nat. Rev. Neurosci.* **18,** 115-126 (2017).

3. Reddan, M. C., Lindquist, M. A. & Wager, T. D. Effect size estimation in neuroimaging. *JAMA Psychiatry* **74,** 207-208 (2017).

4. Nieto-Castañón, A. & Fedorenko, E. Subject-specific functional localizers increase sensitivity and functional resolution of multi-subject analyses. *Neuroimage* **63,** 1646-1669 (2012).

5. Yarkoni, T. Big correlations in little studies: Inflated fMRI correlations reflect low statistical power—Commentary on Vul et al. (2009). *Perspect. Psychol. Sci.* **4,** 294-298 (2009).

6. Yarkoni, T., Poldrack, R. A., Van Essen, D. C. & Wager, T. D. Cognitive neuroscience 2.0: Building a cumulative science of human brain function. *Trends Cogn. Sci. (Regul. Ed. )* **14,** 489-496 (2010).

7. Saxe, R., Brett, M. & Kanwisher, N. Divide and conquer: A defense of functional localizers. *Neuroimage* **30,** 1088-1096 (2006).

8. Kriegeskorte, N., Simmons, W. K., Bellgowan, P. S. F. & Baker, C. I. Circular analysis in systems neuroscience: the dangers of double dipping. *Nat. Neurosci.* **12,** 535-540 (2009).

9. Yarkoni, T., Poldrack, R. A., Nichols, T. E., Van Essen, D.,C. & Wager, T. D. Large-scale automated synthesis of human functional neuroimaging data. *Nat. Meth.* **8,** 665-670 (2011).
